# Establishing a Non-Surgical Mouse Model of Laryngopharyngeal Reflux Disease: Acid-Induced Epithelial Disruption and Protective Role of N-Acetylcysteine

**DOI:** 10.3390/cells15020210

**Published:** 2026-01-22

**Authors:** You Yeon Chung, Byoungjae Kim, Juhyun Lee, Sooun Kwak, Mingeun Jung, Yeon Soo Kim, Seung-Kuk Baek

**Affiliations:** 1 Department of Otorhinolaryngology-Head and Neck Surgery, College of Medicine, Korea University, Seoul 02842, Republic of Korea; 2 Neuroscience Research Institute, College of Medicine, Korea University, Seoul 02842, Republic of Korea

**Keywords:** laryngopharyngeal reflux disease, non-surgical mouse model, epithelial barrier disruption, E-cadherin, MMP-7, ROS–ERK–c-Jun signaling, N-acetylcysteine, mucosal protection

## Abstract

Laryngopharyngeal reflux disease (LPRD) results from the retrograde flow of gastric contents into the upper aerodigestive tract, causing epithelial injury. Progress in its management has been limited by the lack of objective biomarkers and reproducible in vivo models. This study aimed to establish a chronic, non-surgical mouse model of LPRD and to investigate the protective effect of N-acetylcysteine (NAC). Female C57BL/6 mice were randomly assigned to three groups: control (standard drinking water), study (acidified water, pH 3.0, for 12 weeks), and treatment (acidified water for 12 weeks plus NAC supplementation during the final 4 weeks). Body weight, food intake, and water consumption were monitored weekly. Pharyngeal tissues were analyzed by immunohistochemistry and Western blotting. Chronic acid exposure resulted in loss of membrane-localized E-cadherin, cytoplasmic redistribution, and upregulation of matrix metalloproteinase-7 (MMP-7). These molecular alterations were accompanied by enhanced phosphorylation of ERK and c-Jun, consistent with activation of the ROS–ERK–c-Jun signaling pathway. NAC supplementation was associated with partial restoration of E-cadherin, reduced MMP-7 expression, and attenuation of ERK/c-Jun phosphorylation. No systemic toxicity or weight loss was observed, indicating good tolerability of the model. This non-surgical ingestion-based model faithfully recapitulates key epithelial features of LPRD and provides a feasible platform for mechanistic investigation and exploratory therapeutic studies. NAC may exert protective effects against acid-induced epithelial injury in this model.

## 1. Introduction

Laryngopharyngeal reflux disease (LPRD) is a chronic and frequently treatment-refractory disorder caused by the retrograde flow of gastric contents into the laryngopharynx, resulting in mucosal irritation, epithelial disruption, and symptoms such as throat clearing, hoarseness, and globus sensation. Despite growing clinical recognition, diagnosis and management remain challenging because of the absence of universally accepted biomarkers and the heterogeneous response to standard therapies. Acid has traditionally been considered the principal injurious agent, but bile acids, pepsin, and trypsin also contribute to epithelial injury, and even weakly acidic refluxates are sufficient to provoke damage [1,2]. Proton pump inhibitors (PPIs), the most widely prescribed therapy, provide at best modest benefits, with randomized trials and meta-analyses often showing no superiority over placebo in patients with persistent throat symptoms [3,4]. Moreover, concerns regarding long-term PPI use—including potential associations with gastric cancer after Helicobacter pylori eradication—have tempered enthusiasm for chronic therapy [5,6,7]. These limitations highlight the urgent need for alternative therapeutic approaches grounded in disease biology.

Recent mechanistic studies implicate epithelial junctional disruption and protease-driven remodeling as central processes in LPRD. Clinical biopsies reveal reduced E-cadherin expression in LPR mucosa with preserved β-catenin [8,9], while in vitro work shows that acid exposure induces matrix metalloproteinase-7 (MMP-7) via a ROS–ERK–c-Jun cascade, leading to E-cadherin cleavage [10,11]. Independent studies corroborate MMP-7 as a key mediator of epithelial barrier remodeling, driving E-cadherin shedding and β-catenin disorganization [12,13]. These epithelial alterations are closely linked to oxidative stress-responsive signaling pathways reported in reflux-related epithelia, including reactive oxygen species-dependent MAPK cascades and inflammatory mediators such as iNOS/NO signaling [14,15,16].

Despite increasing insights into the molecular mechanisms underlying epithelial injury, translation of these findings into effective therapies has remained limited. In clinical practice, LPRD is frequently diagnosed based on symptom indices or empirical PPI trials, both of which lack specificity and predictive value [3,4]. This disconnect between mechanistic understanding and clinical management underscores the need for experimental models that enable simultaneous evaluation of epithelial injury, signaling pathways, and therapeutic modulation in a controlled in vivo setting.

Animal models of reflux have traditionally relied on invasive surgical procedures such as esophagogastroduodenal or esophagoduodenal anastomoses, which are associated with substantial perioperative morbidity, malnutrition, and severe pan-esophagitis [17,18]. Alternative approaches, such as catheterized laryngeal exposure in anesthetized rats, provide proof of principle but are limited by variability in pH, pepsin concentration, and delivery methods [19,20]. Recently, we reported that dietary paradigms, such as overeating-induced reflux in mice, have demonstrated the feasibility of non-surgical behavioral models in gastroesophageal reflux disease (GERD) [21]. However, such ingestion-based models inevitably simplify the complex composition of human refluxate, which may include pepsin, bile acids, and mechanical reflux components, and therefore represent only selected aspects of reflux-induced injury rather than the full clinical spectrum of human LPRD. Accordingly, findings derived from ingestion-based paradigms should be interpreted within the context of acid-specific epithelial injury rather than as a comprehensive model of all reflux-related mechanisms.

In this study, we established a non-surgical, ingestion-based mouse model of LPRD using acidified drinking water to induce chronic pharyngeal epithelial injury. This model addresses the limitations of prior invasive methods by providing a reproducible, physiologically relevant, and ethically feasible platform. Furthermore, we tested the potential protective effect of N-acetylcysteine (NAC), an antioxidant/mucolytic with preliminary evidence of benefit in LPR when used as an adjunct to PPI therapy [22]. Given that acid exposure activates MAPK signaling pathways in reflux-related epithelia, antioxidant modulation of ERK and AP-1-associated signaling represents a biologically plausible therapeutic strategy [23,24]. By interrogating canonical mechanistic endpoints—including E-cadherin localization, MMP-7 expression, and ERK/c-Jun activation—we demonstrate the biological plausibility of this ingestion-based model and propose its utility as a practical preclinical platform for therapeutic evaluation.

## 2. Materials and Methods

### 2.1. Animal Model and Experimental Design

Five-week-old female C57BL/6 mice (n = 12) were obtained from ORIENTBIO (Seongnam, Korea) and maintained under specific pathogen-free (SPF) conditions in a controlled environment (22 ± 2 °C, 50–60% humidity, 12-h light/dark cycle). All experimental protocols were approved by the Institutional Animal Care and Use Committee (IACUC) of Korea University (Approval No. KOREA-2021-0196) and conducted in accordance with institutional and national guidelines for animal research.

**Figure 1:**
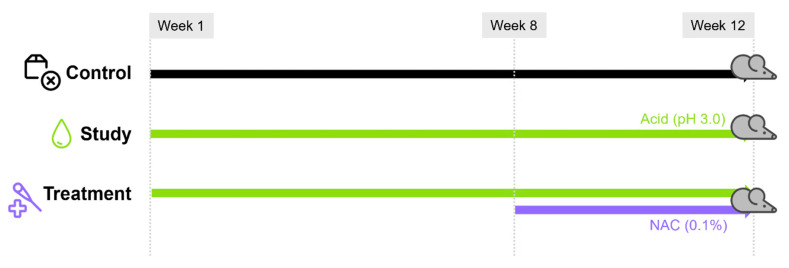
Experimental design of the LPRD mouse model. Schematic illustration of the experimental groups. The control group received standard drinking water for 12 weeks, the study group received acidified water (pH 3.0) for 12 weeks, and the treatment group received acidified water for 12 weeks with additional N-acetylcysteine (NAC, 0.1%) administered during weeks 8–12.

Mice were randomly assigned to three groups (n = 4 per group; Figure 1). Group allocation was performed using a simple randomization procedure by an investigator blinded to downstream analyses. The sample size was determined based on ethical considerations to minimize animal use while retaining sufficient tissue for both histologic and biochemical analyses, rather than on a prespecified statistical power calculation. The control group received standard drinking water for 12 weeks. The study group (reflux group) was provided with acidified water (pH 3.0, adjusted with 0.1 M hydrochloric acid) for 12 weeks. The treatment group received the same acidified water throughout the experiment and additionally N-acetylcysteine (NAC) at a final concentration of 0.1% (*v*/*v*), a dose selected based on previous studies demonstrating antioxidant efficacy and tolerability during chronic administration. NAC (Mucomyst^®^, 20% solution; purchased from Boryung, Seoul, Republic of Korea) was dissolved in the drinking water and administered during the final four weeks (weeks 8–12). All drinking solutions were replaced every 2–3 days, and pH was monitored twice weekly using test strips to ensure stability. NAC-containing water was wrapped in aluminum foil during storage to minimize light exposure.

### 2.2. Monitoring of Physiological Parameters

Throughout the 12-week experimental period, body weight, food intake, and water consumption were monitored weekly to evaluate the general health status of the mice and to identify any potential adverse effects of acidified water or NAC administration. Body weight was recorded individually for each mouse, whereas food and water consumption were measured at the cage level and normalized to an average daily intake per mouse.

### 2.3. Tissue Collection

After the 12-week experimental period, all mice were euthanized under isoflurane inhalation anesthesia followed by CO_2_ exposure. Pharyngeal tissues were harvested and processed for histological and biochemical analyses. For Western blotting, mucosal layers corresponding to the oropharynx and hypopharynx were carefully scraped, snap-frozen in liquid nitrogen, and stored at −80 °C until analysis. For immunohistochemistry, tissues encompassing the oropharyngeal and hypopharyngeal regions were fixed in 4% paraformaldehyde, embedded in paraffin, and sectioned for subsequent staining.

### 2.4. Immunohistochemistry (IHC)

Paraffin-embedded tissue sections (4 µm) were deparaffinized, rehydrated, and incubated with 0.3% hydrogen peroxide (H_2_O_2_) in distilled water for 30 min to block endogenous peroxidase activity. Heat-induced antigen retrieval was then performed using citrate buffer (pH 6.0). After blocking, sections were incubated overnight at 4 °C with primary antibodies against E-cadherin (1:5000; Proteintech, Rosemont, IL, USA) or MMP-7 (1:150; Abcam, Cambridge, UK). Subsequently, sections were treated with biotinylated anti-rabbit IgG (H + L) secondary antibody (Vector Laboratories, Newark, CA, USA; E-cadherin: 1:200, MMP-7: 1:500) in PBS for 1 h at room temperature. Antigen–antibody complexes were visualized using the Vectastain ABC Kit (Vector Laboratories) and a 3,3′-diaminobenzidine (DAB) Substrate Kit (Vector Laboratories). After counterstaining with Mayer’s hematoxylin, images were obtained with an Olympus BX51 microscope (Olympus, Tokyo, Japan). Representative images were acquired at ×200 magnification for overall evaluation of staining patterns (particularly MMP-7), while high-magnification images (×400) were used to assess membrane localization and intracellular distribution of E-cadherin.

### 2.5. Western Blot Analysis

Pharyngeal tissues were homogenized in RIPA lysis buffer supplemented with protease and phosphatase inhibitors (Roche, Basel, Switzerland). Total protein concentrations were determined using a BCA assay. For Western blot analysis, protein lysates were mixed with 5× Laemmli buffer at a 2:1 ratio (protein/buffer) and boiled at 95 °C for 10 min to denature proteins. The denatured samples (10 µL per lane) were separated by SDS-PAGE and transferred onto PVDF membranes. After blocking with 5% skim milk, membranes were incubated overnight at 4 °C with primary antibodies against E-cadherin (1:2000; CST), MMP-7 (1:500; Abcam), ERK (1:1000; CST), p-ERK (1:1000; CST), c-Jun (1:1000; CST), p-c-Jun (1:1000; CST), or β-actin (1:2000; Santa Cruz Biotechnology, Dallas, TX, USA) as a loading control. Membranes were then incubated with horseradish peroxidase-conjugated anti-rabbit (1:1000–1:2000; Santa Cruz) or anti-mouse (1:2000; Santa Cruz) secondary antibodies in blocking solution. After washing, protein bands were visualized using an enhanced chemiluminescence (ECL) detection kit (Santa Cruz Biotechnology), and images were captured with a ChemiDoc imaging system (Bio-Rad Laboratories, Hercules, CA, USA). Band intensities were quantified by densitometric analysis using ImageJ v1.50i software (National Institutes of Health, Bethesda, MD, USA), normalized to β-actin, and expressed as fold changes relative to the control group.

### 2.6. Data Analysis

All data are presented as mean ± standard deviation (SD) unless otherwise specified. Values were calculated for all measured parameters using Microsoft Excel. Body weight was recorded individually for each mouse (n = 4 per group per week) to capture subtle inter-animal variations, whereas food and water consumption were measured at the cage level (four mice per cage) and averaged to estimate daily group intake. Because independent replicates were not available for food and water consumption, these values were not subjected to formal statistical testing. Instead, weekly trends in body weight and consumption patterns were plotted to visually assess potential variations or qualitative differences among groups. Statistical tests were not performed for these parameters, as their primary purpose was to confirm the absence of overt toxicity or stress rather than to detect minor differences. For molecular analyses, including Western blot and immunohistochemical data, densitometrically quantified values were analyzed descriptively. Formal statistical hypothesis testing was not performed for these endpoints, as the primary objective of this study was to establish biological plausibility and reproducibility of epithelial injury patterns and signaling alterations in a chronic in vivo model, rather than to assess statistical significance. Figures were generated using GraphPad Prism version 10.4.2 (GraphPad Software, San Diego, CA, USA) and are presented as line graphs.

## 3. Results

### 3.1. Comparable Body Weight Trends Among Experimental Groups

**Figure 2:**
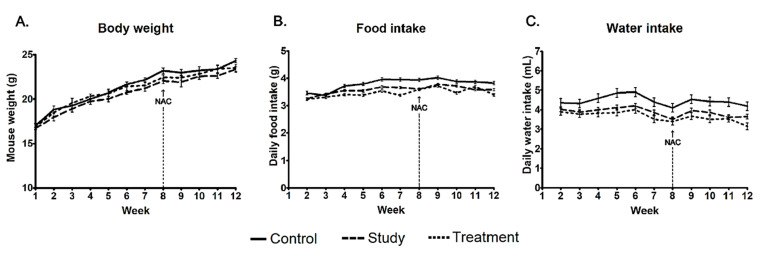
Physiological monitoring over a 12-week experimental period. (**A**) Body weight, (**B**) average daily food intake, and (**C**) average daily water intake were recorded weekly. Vertical dashed lines and arrows denote the initiation of NAC administration in the treatment group at week 8. Data are presented as mean ± SD. Food and water intake were measured at the cage level (four mice per cage).

Body weight was monitored weekly for each mouse to evaluate systemic effects of acidified water and NAC administration (Figure 2A). Mean body weight increased progressively in all groups and remained within a comparable range throughout the 12-week period. Visual inspection of the growth curves did not reveal any consistent group-specific divergence at any time point, suggesting that neither chronic acid exposure nor subsequent NAC supplementation induced overt systemic toxicity or growth impairment.

### 3.2. Numerical Reduction and Partial Recovery of Food and Water Intake

Average daily food (g) and water (mL) intake were assessed weekly to monitor feeding behavior and hydration status (Figure 2B,C). The control group consistently demonstrated slightly higher consumption compared with both acid-exposed groups. Although formal statistical testing was not performed because food and water intake were measured at the cage level, the study and treatment groups showed modest week-to-week fluctuations with consistently lower mean values relative to controls. A noticeable decrease in both food and water intake was observed around week 7, after which the treatment group demonstrated a partial upward trend following the initiation of NAC administration at week 8 (vertical dashed line in Figure 2). These descriptive data are consistent with subtle alterations in feeding behavior during chronic acid exposure, with a modest upward trend observed following NAC supplementation.

### 3.3. Acid-Induced MMP-7 Upregulation and E-Cadherin Loss Mitigated by NAC

**Figure 3:**
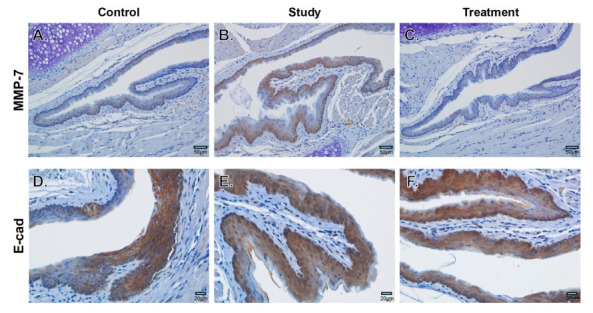
Immunohistochemical analysis of MMP-7 and E-cadherin in pharyngeal epithelium. Representative images of (**A**–**C**) MMP-7 and (**D**–**F**) E-cadherin staining in the control, study, and treatment groups at low (×200) and high magnification (×400). Acid exposure increased cytoplasmic MMP-7 expression and disrupted membrane-localized E-cadherin, while NAC supplementation reduced MMP-7 expression and partially restored E-cadherin. Based on qualitative observation of representative images, restoration of membrane-localized E-cadherin appeared to be more evident in the basal epithelial layer. Scale bars are indicated.

Immunohistochemical analysis of pharyngeal tissues demonstrated distinct epithelial staining patterns across groups. In the control group, MMP-7 expression was minimal, with weak cytoplasmic staining confined to basal epithelial layers, consistent with low proteolytic activity (Figure 3A). E-cadherin was strongly localized at cell membranes, reflecting well-preserved intercellular junctions and intact epithelial architecture (Figure 3D). In contrast, the study group exhibited focally increased MMP-7 expression, characterized by strong cytoplasmic staining throughout the epithelial layers, indicative of enhanced proteolytic activity and tissue remodeling (Figure 3B). This was accompanied by localized disruption of E-cadherin, with loss of membrane staining and diffuse cytoplasmic redistribution, reflecting impaired epithelial cohesion and compromised structural integrity (Figure 3E). In the treatment group, MMP-7 expression was substantially reduced compared with the study group (Figure 3C), while E-cadherin membrane localization was partially restored, appeared to be more evident in the basal epithelial layer based on qualitative observation, with reduced cytoplasmic mislocalization (Figure 3F). Collectively, these findings indicate that chronic acid exposure is associated with epithelial remodeling through MMP-7-mediated matrix degradation coupled with disruption of cell–cell adhesion, whereas NAC supplementation mitigates these alterations by suppressing proteolytic activity and preserving epithelial integrity.

### 3.4. Modulation of Epithelial Markers and MAPK Pathway by Acid and NAC

**Figure 4:**
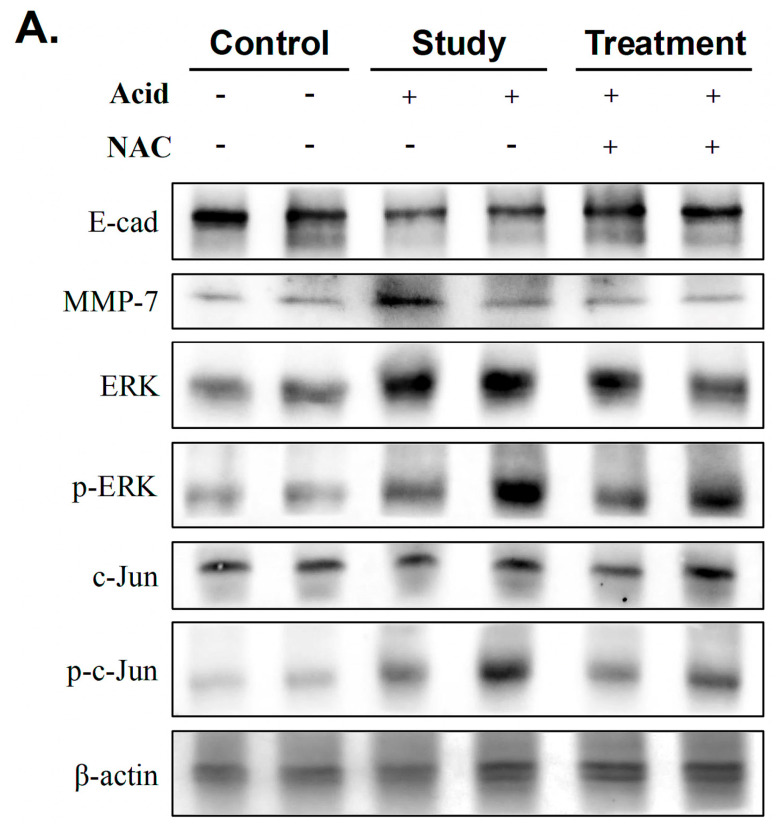

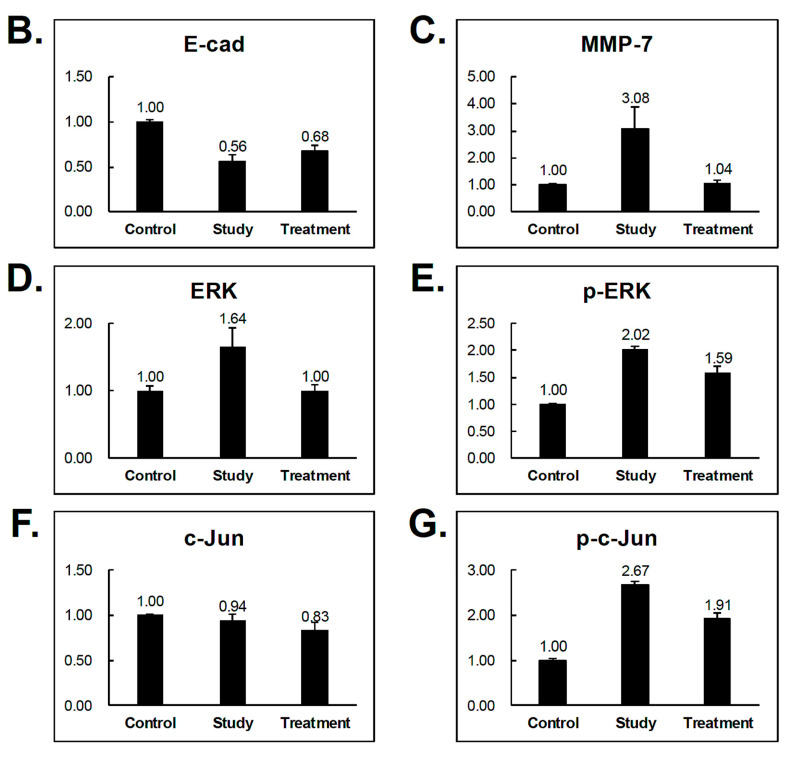
Western blot analysis of epithelial markers and signaling proteins. (**A**) Representative blots of E-cadherin (E-cad), MMP-7, ERK, phosphorylated ERK (p-ERK), c-Jun, and phosphorylated c-Jun (p-c-Jun), with β-actin used as a loading control. (**B**–**G**) Densitometric quantification of band intensities normalized to β-actin. Values are expressed relative to the control group (set to 1.00) and are presented as mean ± SD.

Western blot analysis supported the immunohistochemical findings, revealing distinct protein expression profiles across the three groups (Figure 4A). Compared with the control group (normalized to 1.00), E-cadherin expression was markedly reduced in the study group (0.56), indicating impaired epithelial adhesion (Figure 4B). NAC administration partially restored E-cadherin levels in the treatment group (0.68), consistent with a protective effect on epithelial barrier integrity. In contrast, MMP-7 expression was substantially upregulated in the study group (3.08 relative to control), reflecting active tissue remodeling and degradation, whereas NAC supplementation reduced MMP-7 expression to near-baseline levels (1.04), consistent with inhibition of acid-induced protease activation (Figure 4C).

Analysis of signaling proteins further demonstrated that acid exposure activated stress-responsive pathways. Phosphorylated ERK and c-Jun levels increased to 2.02 and 2.67, respectively, in the study group (Figure 4E,G), confirming activation of ERK and AP-1 signaling under acid-induced stress. In the treatment group, phosphorylation levels were reduced to 1.59 (p-ERK) and 1.91 (p-c-Jun), indicating that NAC attenuated these stress-related signaling cascades. Total ERK and total c-Jun expression remained relatively stable across groups, with only minor variations, which were less pronounced than those observed in their phosphorylated counterparts (Figure 4D,F). Collectively, these molecular data indicate that acid exposure activates stress-responsive signaling pathways, upregulates MMP-7, and disrupts epithelial barrier-associated proteins, whereas NAC supplementation effectively counteracts these alterations, highlighting its protective role against acid-induced molecular stress in pharyngeal tissues.

## 4. Discussion

**Figure 5:**
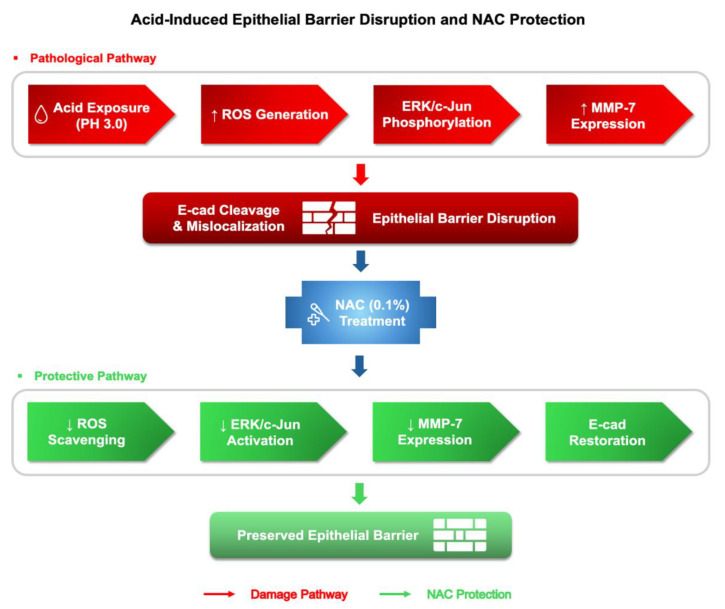
Proposed mechanism of acid-induced epithelial barrier disruption and NAC-mediated protection. Schematic summary of pathological (red) and protective (green) pathways. Acid exposure activates the ROS–ERK–c-Jun–MMP-7 cascade, leading to E-cadherin cleavage and epithelial barrier disruption. NAC suppresses this cascade, reduces MMP-7 expression, and restores E-cadherin localization, thereby preserving epithelial integrity. Arrows indicate the direction of change or signaling flow: upward arrows (↑) denote increased activity or expression, downward arrows (↓) denote decreased activity, red arrows represent the pathological damage pathway, and green arrows indicate NAC-mediated protective effects.

Acidified water ingestion, used to mimic the mucosal irritation characteristic of LPRD, induced distinct epithelial alterations in the murine laryngopharynx. In particular, the epithelium exhibited localized loss of membrane-associated E-cadherin and marked upregulation of MMP-7, indicating disruption of epithelial cohesion and activation of proteolytic pathways. NAC supplementation partially restored epithelial integrity in a region-specific manner, with more discernible changes observed in the basal epithelial layer based on qualitative assessment, supporting its protective effect against chronic acid exposure. These findings are schematically summarized in Figure 5, which illustrates the acid-induced ROS–ERK–c-Jun–MMP-7 cascade leading to E-cadherin cleavage and the partial attenuation achieved by NAC.

Collectively, these results support the biological plausibility of our non-surgical ingestion-based reflux model and are consistent with clinical reports demonstrating decreased E-cadherin [8,9] with preserved β-catenin [9] in LPRD mucosa, as well as mechanistic studies implicating MMP-7-mediated E-cadherin cleavage [10,13] and acid-triggered ROS–ERK–c-Jun signaling upstream of MMP-7 [11].

Traditional reflux models have relied on surgical manipulations such as esophagogastroduodenal anastomosis (EGDA) to induce chronic reflux [17,18]. Although physiologically relevant, these models are invasive, technically demanding, and often accompanied by perioperative stress and limited survival, which restrict their suitability for long-term mechanistic or pharmacologic investigations. In contrast, non-surgical paradigms minimize procedural variability and enable repeated, controlled exposure. Recent non-surgical GERD studies in mice using overeating-induced reflux have demonstrated the practicality and reproducibility of ingestion-based approaches [21]. Building upon this concept, our acid-only ingestion model isolates acidic exposure as the primary causative factor, allowing for tissue alterations and pharmacologic responses to be attributed specifically to epithelial–acid interactions rather than surgical artifacts. Body weight remained stable across all groups throughout the 12-week period, and group differences in food and water intake were modest and descriptive rather than statistically evaluated. A transient decrease in intake was observed around weeks 6–8, particularly in acid-exposed groups, with partial recovery in the treatment group following initiation of NAC administration. The combination of long-term physiological stability and non-invasiveness supports the model’s suitability for chronic mechanistic or screening studies, in contrast to surgical reflux models that require laparotomy and mucosa-to-mucosa anastomoses and are associated with perioperative morbidity, mortality, and, in some procedures such as EGDA, nutritional or severe inflammatory complications [17,18].

Histological and immunoblot analyses demonstrated junctional disruption, evidenced by loss of membrane E-cadherin, and epithelial remodeling characterized by MMP-7 induction—both of which were partially reversed by NAC. Notably, E-cadherin loss was not diffuse throughout the mucosa but localized to specific regions, consistent with the ingestion-based exposure model, in which acid transiently contacts the pharyngeal surface during swallowing. Four weeks of NAC supplementation produced limited yet discernible recovery, most apparent at the basal epithelial layer, suggesting a gradient of repair. The present study was designed to evaluate post-injury recovery rather than prophylactic intervention, and longer or earlier NAC administration may yield more pronounced protective effects. These findings parallel patient biopsy data showing reduced E-cadherin expression in LPRD mucosa. MMP-7 was selected as a biomarker due to its established role in mediating E-cadherin cleavage and epithelial remodeling [10,11,12,13]. Mechanistically, acidic exposure of primary human pharyngeal epithelial cells activates a ROS–ERK–c-Jun–MMP-7 cascade that induces E-cadherin cleavage, a process inhibited by NAC. The convergence between our in vivo findings and these cellular mechanisms strengthens the biological plausibility of NAC-mediated protection. Acid exposure induced phosphorylation of ERK and c-Jun, and this activation was effectively suppressed by NAC. This pattern identifies the ERK–AP-1 axis as a central mediator of reflux-associated epithelial disruption. Consistent with this, previous studies in esophageal epithelial cells have shown that acid or acidic bile salts activate MAPK (ERK/p38/JNK) and AP-1 signaling pathways, promoting inflammatory responses such as IL-8 induction and cellular proliferation [23,24,25]. Laryngeal injury models further support the contribution of oxidative stress mechanisms to reflux-related epithelial responses [19,20].

NAC protected against acid-induced epithelial disruption by attenuating ROS–ERK–c-Jun activation, downregulating MMP-7, and partially restoring epithelial integrity in a region-specific manner. However, given the pleiotropic antioxidant effects of NAC and the limitations of in vivo animal models, direct causal inhibition of MMP-7 cannot be conclusively established in the present study. Comparative studies using selective MMP-7 inhibitors will be required to further delineate the specific contribution of MMP-7 suppression. Although the recovery was incomplete, these findings suggest that NAC alone may provide partial protection against acid-induced epithelial injury, supporting its potential therapeutic relevance independent of PPIs. While previous clinical studies have primarily evaluated NAC in combination with PPIs [22], the present results provide experimental evidence that NAC monotherapy can also mitigate reflux-associated epithelial injury, thereby expanding its potential clinical relevance. Given its favorable safety profile, NAC warrants further investigation as a mucosal protectant under reflux-like conditions. These results are conceptually consistent with the broader oxidative and inflammatory signaling cascades reported in reflux models, including iNOS/NO and NF-κB/AP-1 pathways [14,15,16], and align with stress-response convergence upstream of MMP-7. This acid-only platform is thus well suited for preclinical screening of selective inhibitors targeting MMP-7 and MAPK/AP-1 signaling [13,23,25], as well as for biomarker translation, since salivary MMP-7 has demonstrated diagnostic potential in LPRD [26]. Importantly, NAC itself serves as a proof-of-concept protective agent, validating MMP-7 as a potential therapeutic target. Building on this foundation, the acid-only ingestion model provides a practical and reproducible platform for preclinical testing of MMP-7-directed therapies and for developing additional intervention strategies. Altogether, these directions highlight the promise of this acid-only ingestion model as a practical platform for mechanistic and therapeutic studies, though its translational value will ultimately depend on further validation and refinement.

Although this model does not fully replicate the complete pathophysiology of human LPRD, the epithelial alterations observed here should be interpreted as acid-specific injury rather than a comprehensive representation of all reflux-related insults. First, the model simplifies reflux to acid exposure alone and does not incorporate other injurious components such as bile salts, pepsin, or mechanical stress; therefore, the epithelial alterations observed here may represent only part of the broader reflux-associated injury spectrum. Second, because NAC treatment was administered only during the final four weeks, the degree of epithelial restoration was necessarily partial, and longer treatment durations may further clarify the therapeutic window. Third, signaling analyses were performed at a single terminal time point, limiting the interpretation of temporal dynamics. Because the animal experiments had already been completed at the time of analysis, and intermediate sacrifices would raise ethical concerns regarding additional animal use, longitudinal signaling analyses were not feasible in the present study.

Despite these constraints, the observed differences between control, acid-exposed, and NAC-treated groups demonstrate that this ingestion-based paradigm is effective for isolating acid-specific epithelial effects and for validating the protective potential of NAC. Notably, the non-surgical design allows for stable environmental control and repeated long-term exposure while avoiding procedural stress, making it a practical and novel in vivo platform. This model enabled us to delineate acid-driven epithelial remodeling, characterize MMP-7-mediated E-cadherin disruption, and identify a ROS–ERK–c-Jun axis responsive to antioxidant intervention. Collectively, these strengths support the value of this model as an efficient and informative tool for understanding the biological consequences of chronic acid exposure in LPRD.

## 5. Conclusions

This study established a reproducible, non-surgical mouse model of laryngopharyngeal reflux disease (LPRD) through chronic ingestion of acidified drinking water. The model recapitulated key pathological features of LPRD, including focal loss of membrane-localized E-cadherin, induction of MMP-7, and activation of the ERK–c-Jun pathway. Supplementation with N-acetylcysteine (NAC) partially restored epithelial integrity and attenuated reflux-associated signaling, underscoring its potential as a mucosal protective agent. By combining technical simplicity with translational relevance, this ingestion-based model provides a practical alternative to invasive surgical reflux models. Its stability and reproducibility make it particularly suitable for mechanistic studies and preclinical drug evaluation. Beyond providing proof-of-concept for NAC, this platform enables systematic testing of targeted inhibitors of MMP-7 and MAPK signaling, as well as the exploration of complementary biomarkers and antioxidant-based therapeutic strategies. In conclusion, this non-surgical model may serve as a clinically relevant framework for studying reflux-associated epithelial injury and facilitate the development of novel therapeutic approaches for LPRD.

## Data Availability

All data relevant to the study are included in the article or are available from the corresponding author upon reasonable request.
